# Detecting radio- and chemoresistant cells in 3D cancer co-cultures using chromatin biomarkers

**DOI:** 10.1038/s41598-023-47287-2

**Published:** 2023-11-24

**Authors:** Tina Pekeč, Saradha Venkatachalapathy, Anne R. Shim, Daniel Paysan, Michal Grzmil, Roger Schibli, Martin Béhé, G. V. Shivashankar

**Affiliations:** 1 Laboratory for Nanoscale Biology, Paul Scherrer Institute, Villigen, Switzerland; 2 Center for Radiopharmaceutical Sciences, Paul Scherrer Institute, Villigen, Switzerland; 3 Department of Health Sciences and Technology, ETH Zürich, Zürich, Switzerland; 4 Department of Chemistry and Applied Biosciences, ETH Zürich, Zürich, Switzerland

**Keywords:** Cancer therapeutic resistance, Radiotherapy

## Abstract

The heterogenous treatment response of tumor cells limits the effectiveness of cancer therapy. While this heterogeneity has been linked to cell-to-cell variability within the complex tumor microenvironment, a quantitative biomarker that identifies and characterizes treatment-resistant cell populations is still missing. Herein, we use chromatin organization as a cost-efficient readout of the cells’ states to identify subpopulations that exhibit distinct responses to radiotherapy. To this end, we developed a 3D co-culture model of cancer spheroids and patient-derived fibroblasts treated with radiotherapy. Using the model we identified treatment-resistant cells that bypassed DNA damage checkpoints and exhibited an aggressive growth phenotype. Importantly, these cells featured more condensed chromatin which primed them for treatment evasion, as inhibiting chromatin condensation and DNA damage repair mechanisms improved the efficacy of not only radio- but also chemotherapy. Collectively, our work shows the potential of using chromatin organization to cost-effectively study the heterogeneous treatment susceptibility of cells and guide therapeutic design.

## Introduction

Intra-tumor heterogeneity in cell states has been a major hurdle to the successful treatment of cancer^1^. The sources of such cell-to-cell variability include the intrinsic heterogeneity in cell states as characterized by multiple genetic mutations and chromosome copy number variations^2,3^, as well as spatial variability in extrinsic microenvironmental factors such as tissue stiffness^4^ and hypoxia^5^. These factors affect cellular structure, function, and hence their response to external stimuli such as therapeutic agents. Therefore, despite the seminal advancements made in developing novel targeted therapies for cancer, a subpopulation of cancer cells often persists after treatment, leading to relapse^6–9^. Hence, there have been multiple efforts made to isolate and characterize these therapy resistant/persistent subpopulations, primarily using single cell RNA sequencing and proteomic approaches^10,11^. These efforts have revealed the existence of multiple clonal subpopulations in cancers which continue to evolve during and after treatment^12^. However, a biomarker that provides a quantitative, robust identification of these cancer subpopulations either pre- or post-treatment is still missing.

To improve treatment prognosis, targeted therapies and combinatorial treatments are being developed to also target these therapy-resistant subpopulations. One such treatment modality that does this and has been shown to be efficacious for the treatment of certain cancer types (e.g. neuroendocrine tumors and prostate cancer)^13^ is targeted radionuclide therapy (TRT). TRT is a type of radiation therapy in which a radionuclide (α or β^−^-particle emitter) is conjugated to a cancer cell-targeting molecule, such as a peptide or monoclonal antibody, which binds to a specific protein overexpressed on cancer cells. This monoclonal antibody or peptide ligand not only allows the radiation-emitting radionuclide to be targeted specifically to cancer cells with the protein expression, but also irradiates cancer cells without protein expression (“crossfire effect”) and may limit radiation-induced harm to non-cancerous cells which do not express this receptor. Recent literature suggests that more successful treatment outcomes can arise by combining therapy that is targeted towards cancer cells with adjuvant treatments that target modulators of important cellular functions, such as chromatin organization^14,15^, DNA damage/repair pathways^16^, cell proliferation^17,18^, and/or extracellular matrix organization^19^. To maximize the success of such treatment models, which are subject to multiple ongoing clinical trials^20,21^, it is important to quantitatively characterize treatment-resistant cellular phenotypes to identify which cellular features allow for maximal escape from treatment.

Recent studies have shown that chromatin structure is a functional integrator of the extracellular microenvironment and transcriptional response, thereby suggesting that the chromatin architecture could provide a valuable biomarker to identify cell populations which can evade treatment^22–26^. Towards this, sequencing-based approaches and conformation capture technologies, high resolution images of DNA can be used to quantitatively describe chromatin organization. Such images have been used along with machine learning methods to discriminate cell types, cancer stages, as well as cancer progression^23,25,27^. Importantly, a recent study has developed methods of inferring gene expression from images of DNA in single cells and vice versa^26^. Collectively, these results highlight the rich information present in the chromatin structure that can reveal cell function/dysfunction and that is cost- and time-efficiently captured using imaging data. We thus hypothesize that the organization of the chromatin within the nucleus could serve as a robust and sensitive biomarker for characterizing cell phenotypes which exhibit different responses to the same therapeutic agent.

Herein, we used an engineered, 3D-tissue model of skin cancer to characterize the variability in cellular response and treatment efficacy of TRT using cost-efficient fluorescent imaging and machine learning. For our treatment model, we used [^177^Lu]Lu-PP-F11N, a radiolabeled (with Lutetium-177) analog of minigastrin PP-F11N, derived from a small peptide hormone that selectively binds to the cholecystokinin B receptor (CCKBR). CCKBR is a G-protein coupled receptor which has been shown to be upregulated in multiple cancers and is thus a promising target of TRT^28–30^. Our results demonstrate that TRT does target cells with the CCKBR receptor, causing decreased spheroid area, increased DNA damage, and increased cell death in cells that express the CCKB receptor. Interestingly, we observed a heterogeneous response of the cells to the treatment, which can be characterized by the amount of DNA damage and cell death markers in the nucleus and is also reflected in the morphological and chromatin organizational phenotypes of the cells. These chromatin states are native to the cell (not resultant of treatment) and in fact could be used to predict the cell’s susceptibility to radiotherapy prior to treatment. Further, the cells which are less able to evade treatment have less condensed chromatin, and upon treating cells with combinatorial therapies that decrease chromatin compaction, the cells are more susceptible both to radionuclide treatment and to chemotherapy. Taken together, our results highlight that the information contained in high-resolution chromatin images can be mined for chromatin conformational information that predicts therapy outcomes and guide the design of novel and improved therapy models.

## Results

### Engineered 3D in vitro skin cancer model to study the efficacy of radiotherapy

**Figure 1:**
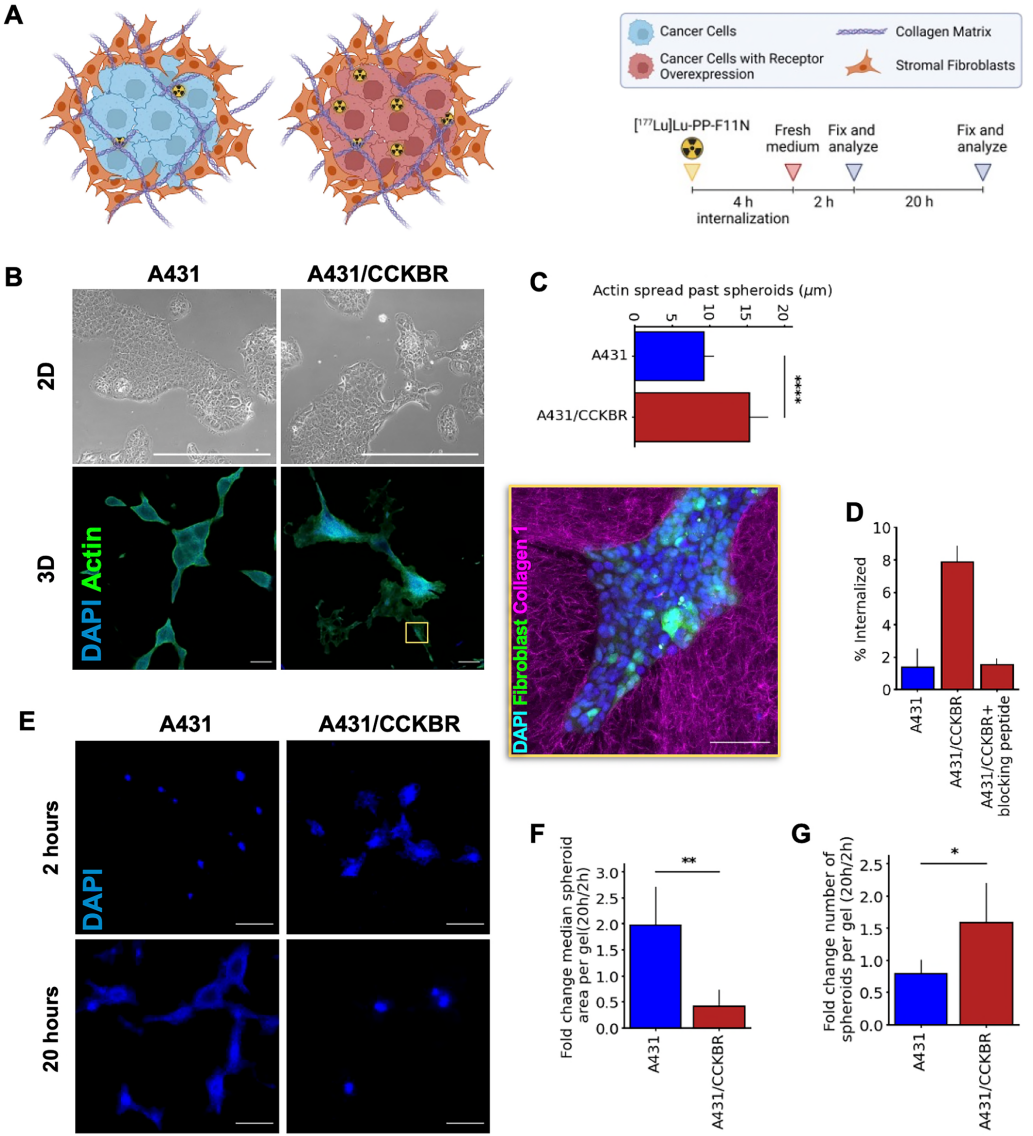
Engineered 3D in-vitro skin cancer model to study the efficacy of radiotherapy. (**A**) A schematic representation of the model system and radioligand treatment protocol (created with BioRender.com). (**B**) Upper: Phase contrast images of A431 and A431/CCKBR cell lines in 2D cultures (scale bar: 500 μm). Lower: Spheroids of A431 and A431/CCKBR cell lines cultured in 3D collagen gel (scale bar: 500 μm). Nuclei are labeled with DAPI (blue) and actin (green) is labeled with ActinGreen. Inset: high resolution (40 ×) image of the cells embedded in a collagen gel (scale bar: 100 μm). Nuclei are labeled with DAPI (blue), fibroblasts (green) are labeled with CellTracker Green and collagen (magenta). (**C**) A431/CCKBR cells have a statistically higher (p = 1.95 × 10^−19^, Welch’s *t* test) actin spread past the edges of the spheroids than A431 cells, demonstrating that A431/CCKBR cells have a more migratory phenotype in 3D compared to A431 cells (n = 71, methods shown in Fig. S1C). (**D**) Internalization fraction of the radioligand for A431 cells, A431/CCKBR cells, and A431/CCKBR cells with a blocking peptide. (**E**) Representative images of the cultures 2 and 20 h after treatment. Nuclei are labeled with DAPI (scale bar: 500 μm). (**F**) There is a statistically significant (p = 0.002, Welch’s *t* test) change in median spheroid area from 2 h after treatment to 20 h after treatment for A431 spheroids and A431/CCKBR spheroids. Median area is measured per gel with n = 5 gels. (**G**) There is a statistically significant (p = 0.03, Welch’s *t* test) change in the number of 3D cultured spheroids from 2 h after treatment to 20 h after treatment for A431 spheroids and A431/CCKBR spheroids. Spheroid number is measured per gel with n = 5 gels.

We designed a physiologically relevant, 3D co-culture model to characterize (1) the efficacy of TRT against receptor-presenting cancer cells, (2) the safety of TRT for non-receptor presenting stromal cells, and (3) the phenotype of cancer cells that are resistant to TRT, i.e., the therapy-resistant phenotype. For our model to accurately reflect the tumor microenvironment in vivo, the cells were studied in a 3D environment and were co-cultured with primary fibroblasts to capture the crosstalk between cancer and stromal cells (Fig. 1A). The novelty of our approach lies in the combination of these two factors. The cancer cells in our 3D co-culture model are A431 cells, a skin cancer cell line that was originally isolated from the epidermis of a patient with epidermoid carcinoma. We studied both naive A431 cells, as well as A431 cells that were stably transfected with CCKBR (hereafter referred to as A431/CCKBR) to mimic receptor-presenting tumor cells that exhibit high radioligand uptake^31^ required for our radiotherapy model (Fig. 1B). Both the A431 and the A431/CCKBR cells were grown into spheroids before co-culturing them with human primary dermal fibroblasts (GM09503 cell line) and embedding in a collagen-1 matrix as described in^32^ (Fig. S1,S3).

Importantly, our engineered 3D model more accurately reflects the characteristics of the tumor microenvironment in vivo than a 2D culture model^32^. This is highlighted by the change in morphology exhibited by the cells in 3D. Under 2D culture conditions, A431 and A431/CCKBR cells showed no large-scale differences in nuclear morphology (Fig. 1B). Once embedded in a collagen gel, CCKBR overexpression led to a significant increase in actin spreading by an average of 1.7 folds (Fig. 1C, Fig. S1). This is in line with studies that have shown a link between GPCR signaling and focal adhesion kinase-mediated cell migration^33^, highlighting the importance of physiologically relevant culture models for studying the in vivo characteristics of tumor cells. The average sizes of the spheroids, the number of cells per spheroid, and the number of cells per gel were all similar for the spheroids with A431 cells and the spheroids with A431/CCKBR cells (Fig. S1).

Next, we treated our 3D co-culture model with a radiolabeled analog of minigastrin that selectively binds to CCKBR. We began with a parametric study of [^177^Lu]Lu-PP-F11N specific activities and incubation times to determine the optimal treatment parameters for maximum radionuclide uptake. The highest A431/CCKBR uptake of [^177^Lu]Lu-PP-F11N was achieved at 73 kBq/pmol after 4 h of incubation (Fig. S2); this condition was selected for all subsequent treatment experiments. This 4-h internalization time was followed by an additional incubation of up to 20 h; previous studies have shown that there is not a substantial increase in DNA damage induced by the radionuclide with incubation times longer than 24 h^34^. Both co-cultures containing untransfected A431 cells, as well as A431/CCKBR cells treated with an unlabeled blocking peptide showed a low background uptake of [^177^Lu]Lu-PP-F11N, as expected (Fig. 1D). We observed a 6% receptor-specific internalization of the radiopeptide (Fig. 1D). This receptor-mediated internalization led to an increase in radioactivity of the cultures with A431/CCKBR overexpression compared with those with A431 alone (2.4-fold increase 2 h after treatment, 8.3-fold increase 20 h after treatment, Fig. S2). Altogether, these results demonstrate that our 3D culture model is amenable for CCKBR-specific radioligand therapy and can be used as a screening platform to study the effect of targeted, therapeutic radio-conjugates in an in vitro tissue model.

### Effect of radiotherapy on spheroid size

We first analyzed the effect of radiotherapy treatment on the sizes of the A431 and A431/CCKBR spheroids. We hypothesized that the internalization of the radiopeptide by the A431/CCKBR cells would cause cell death within the spheroids and halt spheroid growth, as opposed to the control A431 spheroids that would continue to grow even after treatment. Indeed, we observed a statistically significant decrease in the mean A431/CCKBR spheroid area 20 h after treatment, compared to 2 h after treatment (Fig. 1E,F). While some of this decrease in spheroid area is due to cell death (see next section), we also observe significantly less A431 spheroids in the gel at 20 h compared to 2 h due to spheroid merging, compared to A431/CCKBR spheroids which increase in number due to fracturing (Fig. 1E,G). Such growth kinetics were also observed in spheroids with primary dermal fibroblasts from another patient (Fig. S1C), indicating the generalizability of our observations.

### Effect of radiotherapy on genome integrity and cell survival

**Figure 2:**
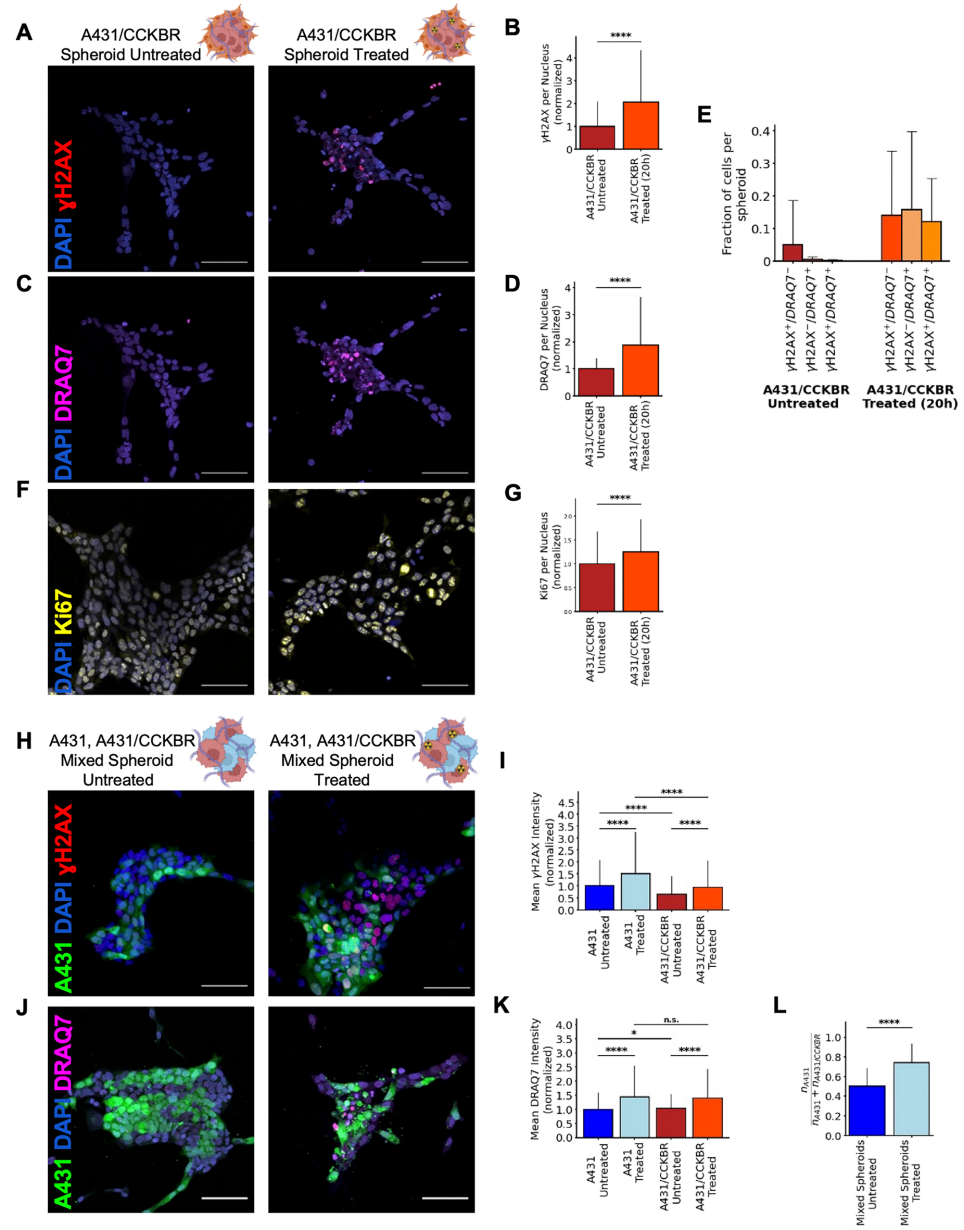
Effect of radiotherapy on genomic integrity and cell survival. (**A**) Fluorescent micrograph of A431/CCKBR 3D co-cultures 20 h after radiolabeled minigastrin treatment and untreated control (scale bar: 100 μm). DNA is stained with DAPI (blue) and DNA damage is labeled with γH2AX (red). (**B**) Sum of γH2AX intensity per nucleus, normalized by the mean γH2AX intensity of untreated A431/CCKBR nuclei. A431/CCKBR spheroids treated with radiotherapy have significantly more DNA damage than untreated A431/CCKBR spheroids (p = 8.99 × 10^−161^, Welch’s *t* test, n = 78 spheroids). (**C**) Fluorescent micrograph of A431/CCKBR 3D co-cultures 20 h after radiolabeled minigastrin treatment and untreated control (scale bar: 100 μm). DNA is stained with DAPI (blue) and cell viability is labeled with DRAQ7 (magenta). (**D**) Mean fluorescence intensity of DRAQ7 per nucleus, divided by the mean DRAQ7 intensity of untreated A431/CCKBR nuclei. A431/CCKBR spheroids treated with radiotherapy have significantly higher levels of cell death than untreated A431/CCKBR spheroids (p = 1.2 × 10^−203^, Welch’s *t* test, n = 78 spheroids). (**E**) Fraction of cells per spheroid which are positive for γH2AX, DRAQ7, or both in the A431/CCKBR 3D co-cultures 20 h after radiolabeled minigastrin treatment or in the untreated controls (n = 78 spheroids). (**F**) Fluorescent micrographs of A431/CCKBR 3D co-cultures 20 h after radiolabeled minigastrin treatment and untreated control (scale bar: 100 μm). DNA is stained with DAPI (blue) and cell proliferation is labeled with Ki67 (yellow). (**G**) Mean Ki67 intensity per nucleus, divided by the mean Ki67 intensity of untreated A431/CCKBR nuclei. Treated A431/CCKBR nuclei display a more aggressive growth phenotype with significantly more Ki67 than untreated nuclei (p = 1.18 × 10^−131^, Welch’s *t* test, n = 17,337 nuclei). (**H**) Fluorescent micrographs of mixed 3D co-cultures (A431:A431/CCKBR in 1:1 ratio) 20 h after radiolabeled minigastrin treatment and untreated control (scale bar: 100 μm). DNA is stained with DAPI (blue), DNA damage is stained with γH2AX (red), and A431 cells are stained with CellTracker Green. (**I**) Mean γH2AX intensity per nucleus, divided by the mean γH2AX intensity of untreated A431 nuclei. Treated A431 and A431/CCKBR nuclei have significantly more DNA damage than untreated nuclei of the same cell types (all p-values < 2.72 × 10^−52^, Welch’s *t* test, n = 21,433 nuclei). (**J**) Fluorescent micrographs of mixed 3D co-cultures (A431:A431/CCKBR in 1:1 ratio) 20 h after radiolabeled minigastrin treatment and untreated control (scale bar: 100 μm). DNA is stained with DAPI (blue), cell viability is stained with DRAQ7 (magenta) and A431 cells are stained with CellTracker Green. (**K**) Mean DRAQ7 intensity per nucleus, normalized by the mean DRAQ7 intensity of untreated A431 nuclei. Treated A431 and A431/CCKBR nuclei have significantly higher levels of cell death marker than untreated nuclei of the same cell types (p = 3.86 × 10^−94^, p = 2.96 × 10^−57^ respectively, Welch’s *t* test, n = 12,288 nuclei). (**L**) The fraction of A431 cells in untreated, mixed spheroids is significantly lower than that of treated, mixed spheroids, indicating significantly more death of A431/CCKBR cells in treated spheroids (p = 8.19 × 10^−9^, Welch’s *t* test, n = 97 spheroids).

Next, we examined if the decrease in spheroid size post-treatment was caused by TRT-induced cell death. TRT with β-emitters (such as Lu-177 used in our study) has been shown to generate reactive oxygen species (ROS), which cause DNA double-stranded breaks, ultimately resulting in cell cycle arrest and cell death^35^. Indeed, when we examined the effect of TRT on the genome integrity of our 3D co-culture spheroids, we found that our skin cancer 3D co-cultures treated with radiotherapy had significantly more (2.1-fold) DNA damage marker γH2AX compared to 3D co-cultures of the same cells without treatment (p < 0.0001, Welch’s *t* test, Fig. 2A,B). This increased damage is also reflected in the viability of the A431/CCKBR cells post-treatment. Cellular expression of the cell death marker DRAQ7 was significantly increased (1.9-fold, p < 0.0001, Welch’s *t* test) in the A431/CCKBR spheroids that received radiotherapy (Fig. 2C,D). These results indicate that radiotherapy as a treatment model can effectively kill cancer cells by inducing DNA damage and ultimately cell death in carcinoma spheroids.

However, although all cells in these carcinoma spheroids overexpressed the CCKBR receptor, not all cells in the spheroid displayed an increase in DNA damage and cell death markers (Fig. S4). While there are significantly higher fractions of cells exhibiting positive γH2AX and DRAQ7 staining in the spheroids which received treatment, the γH2AX and DRAQ7 positive cells in these spheroids still only comprise approximately 30% of the cells in the treated spheroid (Fig. S4). Interestingly, even among these cells which were affected by radiotherapy, we observed a heterogeneous response of the cells to the treatment as characterized by the measured markers for DNA damage and cellular viability (Fig. 2E). In approximately equal ratios we observed cells that were (a) γH2Ax^(+)^/DRAQ7^(+)^, (b) γH2Ax^(−)^/DRAQ7^(+)^ and (c) γH2Ax^(+)^/DRAQ7^(−)^. This divides our treatment-affected cells into three subpopulations: (a) cells which die prior to mounting a DNA damage response, (b) cells which mount a DNA damage response prior to death, and c) cells which are highly damaged but still viable. Interestingly, the cells that are still viable post-treatment display a more aggressive growth phenotype than untreated controls (Fig. 2F); A431/CCKBR cells 20 h post-radioligand treatment express 1.3-fold more proliferation marker (Ki67) than untreated A431/CCKBR cells (Fig. 2G). This is evidence for a highly aggressive, treatment-resistant population of cells. This heterogeneous response and aggressive phenotype is explored further in the next section.

An important feature of TRT is the ability to target cancer cells that express a certain receptor, while causing less damage to surrounding cells which do not express that receptor. To test the ability of our radiotherapy model to exhibit this targeted treatment, we engineered mixed spheroids consisting of equal amounts of A431 and A431/CCKBR cells (Fig. 2F,H,J). While the A431 cells cannot internalize the radiopeptide, both the A431 cells and A431/CCKBR cells exhibit higher levels of γH2Ax and DRAQ7 post-treatment (Fig. 2I,K). This suggests the existence of a “crossfire” effect, whereby tumor cells adjacent to A431/CCKBR cells with internalized radiopeptides are also irradiated, as previously reported for the β-emitter Lu-177 used in our study^36^. Indeed, when we measured the radioactivity of our spheroids after treatment with [^177^Lu]Lu-PP-F11N, A431 cells co-cultured with fibroblasts displayed the least amount of radioactivity, followed by mixed spheroids of A431 and A431/CCKBR cells co-cultured with fibroblasts, and finally A431/CCKBR cells co-cultured with fibroblasts, which displayed the highest radioactivity (Fig. S4). This further supports our findings that there is a “crossfire” effect which increases radioactivity for cancer cells adjacent to A431/CCKBR cells. Contrary to our hypothesis, in mixed spheroids treated with radiotherapy, A431 cells display either comparable or higher levels of γH2Ax and DRAQ7 than A431/CCKBR cells post-treatment (Fig. 2I,K). The reason for this is that A431 cells have a higher survival rate post-treatment. Prior to treatment, A431 cells comprise 50% of the carcinoma spheroids. After treatment, A431 cells comprise 74% of the spheroids, indicating significantly more death of A431/CCKBR cells in treated spheroids (Fig. 2L). Therefore, while the highly affected A431/CCKBR cells die off, the remaining population of A431 cells, while damaged, are still alive. Jointly our results show that the A431 cells which overexpress CCKBR are targeted effectively by our TRT model.

### Nuclear chromatin organization reflects heterogeneous radiotherapy effects and predicts therapy outcomes

**Figure 3:**
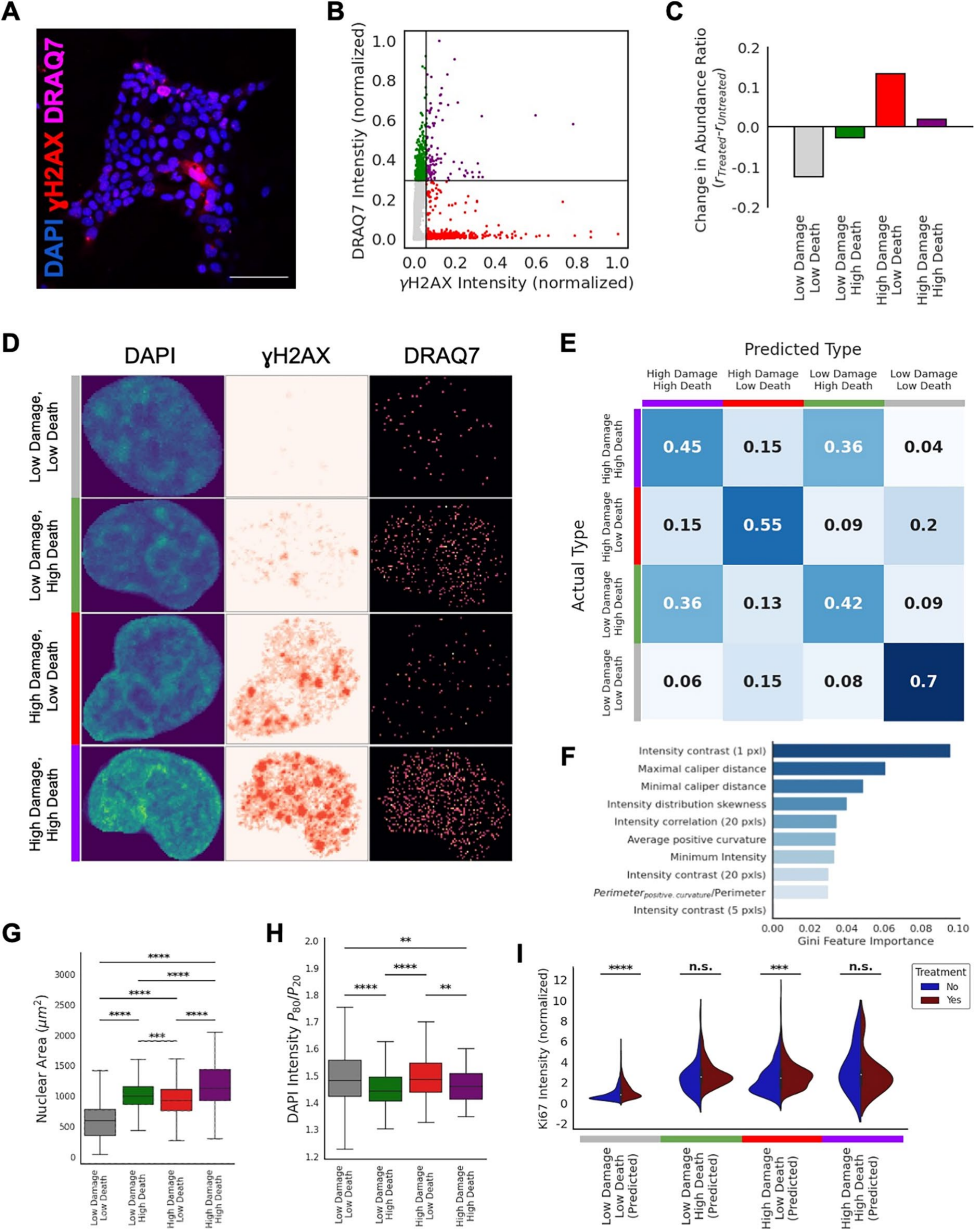
Using nuclear features to predict therapy outcomes. (**A**) Fluorescent micrograph of A431/CCKBR 3D co-culture 20 h after radiolabeled minigastrin treatment (scale bar: 100 μm). DNA is labeled with DAPI (blue), DNA damage is labeled with γH2AX (red), and cell viability is labeled with cell death marker DRAQ7 (magenta). (**B**) A scatter plot of γH2AX intensity against DRAQ7 intensity. Each datapoint represents a single nucleus (n = 5137 nuclei). To demarcate the subpopulations, we identify thresholds based on the 90th percentile of DRAQ7 and γH2AX intensity levels in the A431/CCKBR nuclei in untreated co-cultures (see Fig. S5). The black solid lines denote the thresholds for identifying the subpopulations. (**C**) The change in the abundance of each subpopulation from the untreated co-cultures to the treated co-cultures. Nuclei with low damage are more abundant in the untreated co-cultures, whereas nuclei with high damage are more abundant in the treated co-cultures, regardless of the cell death marker intensity. (**D**) Representative images of a cell from each subpopulation, including its corresponding γH2AX and DRAQ7 images. (**E**) A confusion matrix demonstrating the performance of a Random Forest classifier at distinguishing the subpopulations based on nuclear shape features and chromatin features alone (excluding γH2AX and DRAQ7 intensities). The matrix represents the balanced accuracy after fivefold cross validation, n > 100 nuclei per subpopulation. The Random Forest classifier has an accuracy of 55% at identifying the subpopulation that each nucleus belongs to (based on the nuclear shape and chromatin features), which is more than twofold higher than random chance (25%). (**F**) The top features used by the Random Forest classifier to identify the subpopulations. A complete list of features can be found in Ref.^32^. (**G**) 2D projected nuclear area of the subpopulations. Nuclear area increases with damage and death. Statistics are performed with a Welch’s *t* test, p < 0.0003 for all subpopulations and n > 100 cells for all subpopulations. (**H**) The ratio of the 80th percentile of DAPI intensity to the 20th percentile of DAPI intensity per nucleus, which is a measure of the chromatin condensation. Both damage and death cause decondensation of chromatin. Statistics are performed with a Welch’s *t* test, p < 0.004 for all indicated subpopulations and n > 100 cells for all subpopulations. Statistics which are not indicated are not significant. (**I**) Violinplot of Ki67 intensity by predicted subtype (predicted by the trained Random Forest classifier). Subtypes with low death marker significantly increase their proliferation marker upon treatment, indicating that treatment causes a more aggressive phenotype in cells which are not damaged to the point of death (p < 0.0004, Welch’s *t* test). However, cells with high death marker do not show an increase in proliferation, indicating cell cycle arrest.

The cells in our 3D co-culture spheroids exhibit a heterogeneous response to treatment (Fig. 3A). This heterogeneous response is reflected in the protein markers for DNA damage and cell viability (i.e., treatment does not induce damage and/or death in all cells). Based on these protein markers, we identified four subpopulations of treatment-resistant cells (Fig. 3B, Fig. S5). The largest subpopulation, accounting for 81% of the cells from the untreated spheroids and 69% of cells from the treated spheroids, contained cells that showed low amounts of DNA damage and low DRAQ7 intensity (Fig. S5). The rest of the cells in the spheroid are (similarly as described in the previous section) divided into three subpopulations, which have either high amounts of DNA damage, high amounts of cell death marker, or both (Fig. 3D). While all four subpopulations are present in the untreated spheroids, upon radioligand treatment, a larger fraction of cells belong to the subpopulations which have high DNA damage (Fig. 3C). This further validates the induction of DNA damage by the radiotherapy treatment.

Importantly, not only are the four identified subpopulations distinguishable by protein markers, but they also have distinct morphological and chromatin organizational phenotypes, i.e., chromatin states (Fig. 3D). This is in agreement with previous studies that have established chromatin as a reliable indicator of cell state and cell state transitions in health and disease^23,25,27^. To quantitatively characterize the chromatin states of the individual cells, we measured a number of established nuclear morphology and chromatin organizational features^32^ extracted from corresponding DNA images of the spheroids (see “Methods”, Supplementary Information). We trained a Random Forest classifier on these morphometric and chromatin features and found that the classifier could discriminate between cells of the four subpopulations with a balanced, fivefold cross validation accuracy of 55% (Fig. 3E), which is more than twofold higher than expected by random chance (25%). This highlights that cells with differential response to radiotherapy feature prominent organizational differences in their nuclei and underlying chromatin. Since no information on the protein expression was used by the classifier, this also shows that the amount of γH2Ax and DRAQ7 present in the nucleus, and presumably the ability of these cells to evade therapy, is reflected in the chromatin organization and nuclear morphology of the cell. We found in particular the nuclear size, curvature and chromatin texture differ significantly between the four subpopulations (Fig. 3F). Notably, both an increase in DNA damage and/or death markers lead to an increase in nuclear size (Fig. 3G), perhaps to facilitate DNA repair as seen previously^37,38^. Moreover, both subpopulations that include “High Death” have less condensed chromatin (Fig. 3H). This is likely the source of the classifier’s confusion between the “Low Damage, High Death” and “High Damage, High Death” subtypes (Fig. 3E). Indeed, we find that the subtypes which are most often confused for each other are those which have similar death markers, indicating that that the condensation of the chromatin is tightly linked to cell viability and that cells with decondensed chromatin are less able to evade radiotherapy treatment, i.e., are less treatment resistant. Importantly, the confusion of the cell populations with similar death markers by the model also limits the model’s accuracy as apoptotic cells appear similar in terms of the chromatin organization. Hence, excluding these apoptotic cells from the analysis or focusing on only cells in a specific cell cycle stage is likely to improve the overall accuracy of the model’s performance but is not subject to this work.

Contrary to what would be expected in healthy cells, the cells with low damage and low death markers were seen to be the most proliferative. Using the trained Random Forest classifier, we could predict which cells—based on their nuclear morphometric and chromatin organizational features—belong to the four previously described cell subpopulations associated with different treatment responses (Fig. 3I). This enabled us to also identify these subpopulations in spheroids that are not stained with γH2Ax and DRAQ7, but instead are stained for proliferation (Ki67). For both cells within spheroids treated with radiotherapy and untreated controls, cells with chromatin that reflect the “Low Damage, Low Death” subpopulation shows the lowest amount of the nuclear proliferation marker. This indicates that cells which are more damaged or less viable are bypassing cell cycle checkpoints and become more aggressive. This aggressive phenotype is aggravated upon treatment. Once treated, subtypes which are more viable significantly increase their nuclear proliferation marker (p < 0.0004, Welch’s *t* test). However, cells which are less viable (i.e., feature high levels of the death marker) do not show an increase in proliferation. Interestingly this does not correlate with damage, further suggesting that these cells have bypassed the cell cycle arrest checkpoint, which is associated with increased DNA damage. In fact, the most aggressive phenotype belongs to the “High Damage, Low Death” subpopulation, indicating that these cells are sufficiently damaged to bypass all growth checkpoints, and are the most dangerous, treatment-resistant phenotype for cancer cells (Fig. 3I).

As all four subpopulations are present in both the untreated and treated spheroids, these chromatin states might also be responsible for priming the cells to respond to, and evade, treatment. In fact, our characterization of the chromatin states of these four subpopulations is based on all cells in both our treated and untreated co-culture spheroids. However, when considering only the treated spheroids, the chromatin states which define our four subpopulations are the same (Fig. S5). This indicates that these chromatin states are native to the cell (not resultant of treatment) and in fact could be used to predict the cell’s susceptibility to radiotherapy prior to treatment. Jointly our findings emphasize that different chromatin states are associated with different treatment responses, which ultimately enables the identification of e.g., therapy resistant cells in our tissue model.

### Improving cellular susceptibility to radiotherapy by targeting chromatin structure

The previous results suggest a direct connection between the chromatin states of cells and their response to treatment. We thus hypothesized that altering the chromatin structure of cells would directly affect their response to treatment and treatment efficacy. To test this hypothesis, we compared therapy efficacy in cells which are co-treated with two different compounds which alter the chromatin structure. One of the compounds, Trichostatin A (TSA), is histone deacetylase inhibitor (HDACi), which inhibits formation of heterochromatin and has been shown to increase the effectiveness of radiotherapy^39^. The other compound, KU-55933, is an ataxia telangiectasia mutated (ATM) inhibitor, which prevents DNA damage repair and induces apoptosis^40^. Based on our previous findings, we hypothesized that co-treatment with TSA will increase treatment efficacy by decondensing chromatin, while treatment with KU-55933 will increase susceptibility to treatment by blocking DNA damage repair.

**Figure 4:**
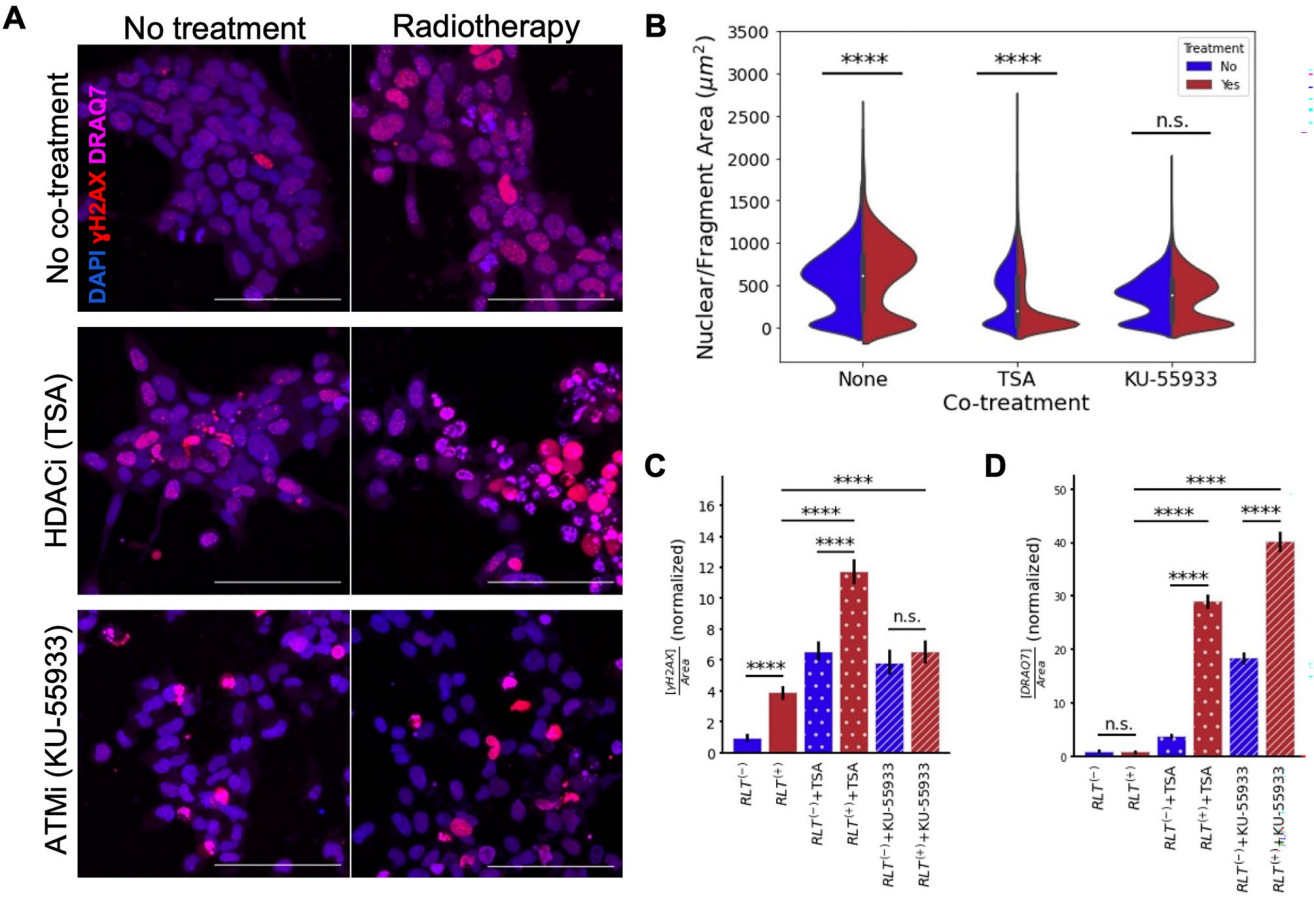
Improving cellular susceptibility to radiotherapy by targeting chromatin structure. (**A**) Fluorescent micrograph of A431/CCKBR 3D co-cultures 20 h after combinatorial treatment (scale bar: 100 μm). DNA is labeled with DAPI (blue), DNA damage is labeled with γH2AX (red), and cell viability is labeled with cell death marker DRAQ7 (magenta). Cultures were treated with radiolabeled minigastrin (right) or untreated controls (left), and combinatorially treated with an HDAC inhibitor (TSA) or an ATM inhibitor (KU-55933). (**B**) The nuclear area of cells or cell fragments. As shown earlier, cells grow after radioligand treatment without combinatorial treatment, in response to increased DNA damage repair. After combinatorial treatment with TSA, cell area decreases significantly compared to spheroids treated with TSA alone (p = 1.0 × 10^−74^, Welch’s *t* test, n = 6374 nuclei) due to cell rupture caused by increased apoptosis. Cell area decreases non-significantly after combinatorial treatment with KU-55933 compared to KU-55933 treatment alone, but decreases significantly compared to untreated spheroids (p = 4.39 × 10^−84^, Welch’s *t* test, n = 5263 nuclei). (**C**) Treatment with TSA and KU-55933 cause increased DNA damage. These treatments alone increase DNA damage even compared to radioligand treatment without combinatorial treatment (p < 3.19 × 10^−6^, Welch’s *t* test, n > 4143 nuclei). Combinatorial treatment of TSA with radiotherapy further causes a significant increase in DNA damage (p = 1.16 × 10^−28^, Welch’s *t* test, n = 6374 nuclei). (**D**) treatment with TSA and KU-55933 cause increased cell death. These treatments alone increase cell death even compared to radioligand treatment without combinatorial treatment (p < 2.48 × 10^−64^, Welch’s *t* test, n > 4143 nuclei). Combinatorial treatment with radiotherapy further causes a significant increase in cell death, both for combinatorial treatment with TSA and KU-55933 (p < 0.000001, Welch’s *t* test, n > 4028 nuclei).

Indeed, we found that both TSA and KU-55933 increase cellular susceptibility to radiotherapy. Qualitatively, combinatorial treatment with these chromatin modifiers and radiotherapy is apparent by the increase in cell rupture and apoptosis (Fig. 4A). This is in contrast with radioligand treatment alone, which increases nuclear size as the cells engage in more DNA damage repair (Fig. 4B). However, when treated with an HDAC inhibitor or ATM inhibitor alone, the area of the remaining nuclei or nuclear fragments decreases significantly compared to treatment with radiotherapy (p < 2.56 × 10^−60^, Welch’s *t* test, n > 4000 nuclei), indicating that the chromatin decondensation caused by TSA and blockage of DNA damage repair caused by KU-55933 activate apoptotic pathways (Fig. 4B). Contrarily, radiotherapy alone damages cells to the point of bypassing apoptotic activation. Treatment with TSA or KU-55933 alone still retains a population of persister cells, which is significantly decreased by combinatorial treatment with TSA and radiotherapy (p = 1.0 × 10^−74^, Welch’s *t* test, n = 6374 nuclei). However, combinatorial treatment of KU-55933 and radiotherapy does not have a significant effect on nuclear sizes, indicating that combinatorial treatment of TSA and radiotherapy has the largest effect on apoptosis.

This finding is supported by the increase in DNA damage and cell death markers in the spheroids treated with combinatorial treatments. While radioligand treatment alone induces fourfold more DNA damage than observed in untreated spheroids, DNA damage increases sixfold when radioligand treatment is used in combination with an ATM inhibitor and increases 12-fold when radioligand treatment is used in combination with an HDAC inhibitor (Fig. 4C). As expected, the ATM inhibitor inhibits DNA damage repair, and the HDAC inhibitor causes chromatin to be more susceptible to DNA damage by opening the structure. Further, this increase in DNA damage leads to over 25-fold more cell death when radiotherapy is combined with one of these chromatin modifiers than when radiotherapy is administered alone (Fig. 4D). Jointly, these findings show that modifying the chromatin structure and repair mechanisms can dramatically increase the effectiveness of radiotherapy. In particular, combinatorial treatment with an HDAC inhibitor causes the most DNA damage and leaves the smallest population of persister cells and is therefore an attractive treatment for increasing radiotherapy efficacy.

### Improving cellular susceptibility to chemotherapy by targeting chromatin structure

**Figure 5:**
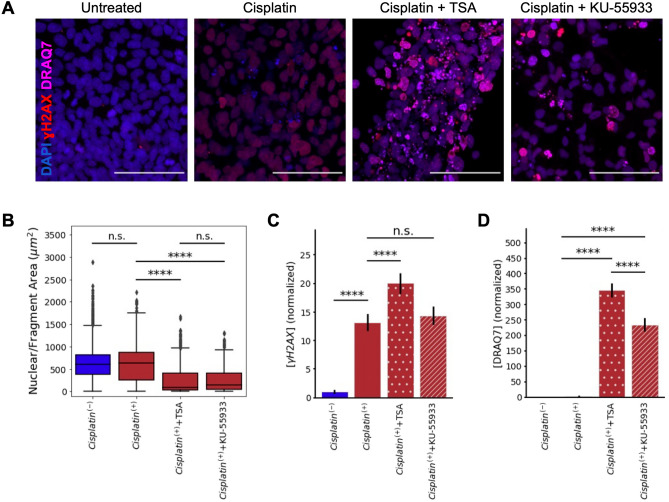
Targeting the chromatin structure also improves cellular susceptibility to chemotherapy. (**A**) Fluorescent micrographs of A431/CCKBR 3D co-culture 20 h after combinatorial chemotherapy treatment with Cisplatin and HDAC inhibitor (TSA) or an ATM inhibitor (KU-55933) (scale bar: 100 μm). DNA is labeled with DAPI (blue), DNA damage is labeled with γH2AX (red), and cell viability is labeled with cell death marker DRAQ7 (magenta). (**B**) The nuclear area of cells or cell fragments. Cell area decreases significantly after combinatorial treatment with TSA or KU-55933 (p < 0.0000001, Welch’s *t* test, n > 5164 nuclei) due to cell rupture caused by increased apoptosis. (**C**) Cisplatin treatment significantly increases DNA damage (p = 5.48 × 10^−82^, Welch’s *t* test, n = 7144). Combinatorial treatment with TSA causes a further increase in DNA damage (p = 3.19 × 10^−10^, Welch’s *t* test, n = 6815), while combinatorial treatment with KU-55933 does not cause a significant increase in damage compared to Cisplatin alone. (**D**) Combinatorial treatment with TSA and KU-55933 cause significantly more cell death than treatment with Cisplatin alone (p < 10^−7^, Welch’s *t* test, n > 5164 nuclei).

We hypothesized that chromatin organization could similarly modulate the efficacy of other therapy models due to the general connection between chromatin organization and e.g., DNA damage response. To test this hypothesis, we treated our 3D co-culture spheroids with Cisplatin, a well-studied chemotherapeutic, both alone and in combination with our chromatin modifying compounds (Fig. 5A). Cisplatin intercalates into DNA and upon replication causes DNA damage leading to cell death^41^. Interestingly, using our model system, we do not see a significant increase of cell death upon Cisplatin treatment, but observe a significant increase in DNA damage (Fig. 5C,D). This finding is in alignment with previous studies that have shown the efficacy of Cisplatin drops in 3D culture as opposed to 2D culture settings^42^. This finding also emphasizes the importance of studying therapy response in complex culture systems such as ours that more accurately mimic the in vivo setting.

In light of these findings, it is especially notable that we observe an increase in DNA damage and cell death when treating our spheroids with Cisplatin in combination with chromatin modifying compounds. The addition of ATM inhibitor KU-55933 to Cisplatin treatment causes a significant increase in cell death but does not increase DNA damage significantly more than Cisplatin treatment alone (Fig. 5C,D). Alternatively, the addition of HDAC inhibitor TSA to Cisplatin treatment causes a significant increase in both DNA damage and cell death (Fig. 5C,D). Both treatments drastically decrease nuclear area, indicating that both inhibition of DNA damage repair and chromatin decondensation cause cells to be more susceptible to chemotherapy treatment, as measured by apoptosis (Fig. 5B). Altogether, these results show that the most significant increase in therapy efficacy is resultant simply by decondensing the chromatin structure. While KU-55933 impairs the DNA damage response, altering this pathway is not necessary to improve therapy efficacy. This can be done simply by altering the chromatin structure.

Jointly, these findings support our hypothesis that the underlying chromatin structure pre-treatment causes cells to be more or less primed to be susceptible to cancer treatments. This highlights the use of chromatin-modifying agents, especially those which induce chromatin decondensation, to increase cancer therapy efficacy.

## Discussion

Intra-tumor heterogeneity is an important source of treatment-resistant subpopulations contributing to relapse^1,6^. Hence, phenotypic characterization of the functionally relevant subsets within the surviving cancer population is required to not only identify these potential persisters pre-treatment, but also identify targets for potential adjuvant therapies. Recent studies have highlighted the physiological relevance of using three-dimensional in vitro models of the tumor microenvironment over conventional 2D culture models^43^. In this study, we used a robust method for generating physiologically relevant, 3D tissue models of cancer spheroids embedded amongst stromal fibroblasts in a collagen matrix^32^. This model system is amenable for high-resolution, single-cell imaging and is conducive for both radionuclide and chemotherapy. Herein we showed that, in line with previous studies done on 2D culture systems, cancer cells in these 3D tissue models internalized the radioligand and induced DNA damage and cell death in cancer cells. While previous studies have reported radiation-indued population regrowth following apoptosis^44^, by co-culturing the receptor-positive and receptor-negative cells, we could measure for the first time the crossfire effect for targeted radioligand therapy in cancer spheroids. Indeed, we showed that both receptor-positive and receptor-negative cells had a significant increase in DNA damage and cell death marker post-TRT treatment. However, treatment increased the ratio of receptor-negative to receptor-positive cells in the spheroids 1.5-fold, indicating more targeted death of receptor-positive cells. This assay shows the importance of TRT and will enable a better understanding of it. However, despite efficient targeting of cancer cells with the radioligand receptor, a subpopulation of viable cancer cells persisted after treatment.

Our finding that different subpopulations of cells respond heterogeneously to treatment opens the possibility of evaluating the biological cause of therapy resistance in detail. Many studies have attempted to identify and characterize such treatment-resistant cells using resource-intensive single cell omics technologies to quantify transcriptomes or proteomes of these cells^45,46^. In this study, we show that such cells can also be identified using chromatin organizational features derived from simple fluorescent DNA images. In particular, we show that the chromatin organization, such as the chromatin condensation, differs significantly between cells that are killed by radiotherapy and those resistant to the treatment, i.e., the persister cell population. Moreover, we show that cells with chromatin organizations that correspond with the chromatin structures seen in cells that have treatment-induced high damage and low death markers take on a more aggressive growth phenotype post-treatment. Therefore, our results suggest that chromatin organization can be used to efficiently identify aggressive persister cells in the heterogeneous cell populations present in complex tumor microenvironments.

Our results establish a connection between native chromatin organizational patterns and the cellular response to radiotherapy treatment. Exploiting this connection, we altered the chromatin organization of our cancer spheroids to increase treatment efficacy. We chose two key modulators of chromatin organization with different mechanistic targets. We investigated combinatorial treatment with an ATM kinase inhibitor (KU-55933), which prevents the activation of DNA damage repair pathways^47,48^, and in turn leads to cell death. We found that indeed the inactivation of DNA repair pathways caused an increase in DNA damage and cell death in conjunction with radiotherapy. We also investigated combinatorial treatment with TSA, an HDAC inhibitor that increases chromatin accessibility. Chromatin architecture is regulated by many molecules, including histone deacetylases (HDACs) that promote chromatin compaction by increasing the electrostatic interactions of DNA and lysines in histone tails^49,50^. By inhibiting HDAC activity, we opened the chromatin structure, thereby making it more permissive for DNA damage induced by the radiation. As expected, this co-treatment significantly increased the effectiveness of radionuclide therapy. Importantly, our results highlight how the chromatin structure itself, not a signaling or repair pathway, regulates cellular receptibility to treatment. This is consistent with our previous findings that use of an HDAC inhibitor can radio-sensitize cancer cells in vivo^51^. We observed similar increases in susceptibility of cells to chemotherapy when treated combinatorially with these chromatin modifying compounds. Therefore, we show that the chromatin structure can prime cells to be protected against a variety of cancer treatments, and modifying this structure can decrease evasion. Importantly, there are many other methods to target the chromatin structure directly or alter the chromatin structure as a consequence of tumor-targeting therapies. For example, EGFR signaling promotes chromatin condensation and anti-EGFR therapies have also been shown to increase the effectiveness of radiation therapies^52^. Therefore, there are many other avenues by which the chromatin structure may be targeted to increase therapy efficacy, highlighting the importance of considering the effect of therapies on the chromatin structure in the design of future therapies.

The work presented herein demonstrates that, in a physiologically relevant 3D co-culture model, the chromatin structure dictates cellular response to therapy. This opens the possibility that such a robust model could provide the groundwork for including other aspects of the tumor microenvironment to further enhance our understanding of how the tumor/stromal crosstalk affects tumor progression. For example, while we include both cancer and stromal cells within this model, conditioning the fibroblasts with cancer signals prior to implantation within the 3D matrix could ensure the existence of cancer associated fibroblasts in the model. In addition, we measure the crossfire effect between A431/CCKBR and A431 cells within mixed spheroids. In this model, these cell types are in contact with each other. However, future studies could measure this crossfire in a trans-well model to investigate how cell signaling specifically affects the chromatin structure of non-CCKBR overexpressing tumor cells. Thus, there are a myriad of opportunities to expand our model system, which will be explored in future studies.

In conclusion, in this study we have presented the nuclear and chromatin architecture as a robust biomarker for characteristic cellular response to therapy and demonstrated how regulators of chromatin structure and function could be potential targets for developing adjuvant treatments to improve the outcome of chemo- and/or radiotherapy. The robustness of this method indicates that our 3D co-culture model captures the in vivo response to treatment and can be used for screening the therapeutic potential of drugs. Importantly, the mechanical state of cells in tissues dictates chromatin structure and gene expression^53^, and tissues of origin have inherent mechanical heterogeneity. This may explain the heterogeneous treatment response, which creates only a subpopulation of persister cells, to a homogeneous extracellular treatment. The biomarkers we propose are tightly linked to the ECM and cellular function and could be valuable resources for designing novel and more efficient therapy options.

## Materials and methods

### Micropatterning

Polydimethylsiloxane (PDMS) stamps were prepared by mixing PDMS elastomer (SYLGARD 184; Dow Corning) with curative-to-precursor in a 1:10 ratio according to the manufacturer’s protocol and by molding the PDMS in microfabricated silicon wafers. Fibronectin solution (10% Fibronectin with 5% Cy5 dye in 1X PBS) was then allowed to adsorb onto the surface of each PDMS stamp under sterile conditions. The coated PDMS stamp was subsequently deposited onto the surface of a hydrophobic dish (Ibidi) to allow the micro features to be transferred. The surface was then treated with 2 mg/ml Pluronic F-127 (Sigma-Aldrich) for 3–5 min to passivate non-fibronectin-coated regions.

### Cell culture

Human epidermoid carcinoma A431 cell line stably overexpressing CCKBR (A431/CCKBR) was developed and kindly provided by Dr. Luigi Aloj^31^. Primary human dermal fibroblasts were sourced from Coriell and were cultured according to their recommendations. GM09503 or GM08401 fibroblasts (p7-12) were cultured in MEM (Life Technologies) supplemented with 10% (vol/vol) fetal bovine serum (GIBCO, Life Technologies), 1% Penn-Strep and 1% NEAA (GIBCO, Life Technologies) at 37 °C in 5% CO_2_. A431 and A431/CCKBR (p7-18) cancer cells were cultured in high glucose DMEM (Life Technologies) supplemented with 10% (vol/vol) fetal bovine serum (GIBCO, Life Technologies) and 1% Penn Strep at 37 °C in 5% CO_2_.

### 3D co-culture of fibroblasts and cancer cell spheroids

Cancer cells (0.25–0.5 × 10^6^) were seeded on fibronectin-coated micropatterns and cultured overnight. After 24 h, the cancer cells formed spheroids, which were washed once with culture media to remove any floating cells. These spheroids were collected and mixed with 3000 fibroblasts, trypsinized from a 70% confluent culture, in a 1 mg/ml rat tail collagen I (ThermoFisher Scientific) solution which was neutralized with 0.1 N NaOH. This solution was allowed to gel at 37 °C for 1–2 h after which 1 ml of culture media was added and the samples were kept for 24–72 h. Mixed spheres were prepared with equal amounts of A431 or A431/CCKBR cells that were mixed prior to adding to the micropattern.

### Radioligand treatment

Radiolabeling of [^177^Lu]Lu-PP-F11N was accomplished at 90 °C for 15 min in 0.4 M ammonium acetate buffer (pH 5.5) with nuclide ([^177^Lu]LuCl_3_ solution; ITM GmbH; Munich, Germany)/peptide (PP-F11N– DOTA-(DGlu)_6_-Ala-Tyr-Gly-Trp-Nle-Asp-Phe; PSL Peptide Specialty Laboratories GmbH; Heidelberg, Germany) ratio of 1:10, 1:30 and 1:100, yielding specific activity of 73, 24.3 and 7.3 MBq/nmol, respectively. The radiochemical purity of [^177^Lu]Lu-PP-F11N was determined by high-performance liquid chromatography (HPLC) using a C18 column, and reached above 98% efficiency (Supplementary Fig. S2B). To determine the cellular uptake, 3D co-cultures were incubated with 25,000 cpm of ^177^Lu-PP-F11N (in DMEM with 0.1% BSA) at standard tissue culture conditions. For blocking experiments, minigastrin (PSL GmbH) was used at 4 µM concentration. After 2 or 4 h incubation, radioactive medium (together with PBS wash) was collected and the cells were subjected to 2 × wash with glycine buffer (pH = 2) for 5 min at RT followed by a dissolving step in 1 M NaOH for 15 min at 37 °C. Collected fractions were measured on a Cobra II Auto-Gamma counter (Packard). Cellular uptake (activity of dissolved cells and glycine wash) was shown as % of total activity (all collected fractions). For imaging and assessment of therapeutic effects, 3D co-cultures were treated with 5 MBq of [^177^Lu]Lu-PP-F11N (73 MBq/nmol) in 1 ml DMEM with 0.1% BSA in a tissue culture incubator. After 4 h incubation, radioactivity-containing medium was removed, and the PBS-washed spheres were further incubated for 2 or 20 h in standard tissue culture conditions.

### Staining of 3D collagen gels

Cocultures stained with DRAQ7 (Thermo Fisher Scientific cat. no. D15105) were cultured in 3 µM DRAQ7 in culture media and kept for 30 min at 37 °C in the cell incubator, protected from light. Afterwards, they were thoroughly washed 3 times 10 min with PBS on a shaker, to exclude the remaining dye from the gels before fixation. Cells stained with CellTracker were treated under 2D culture conditions. Media was removed from the 2D cells and they were washed once with PBS. CellTracker Green CMFDA (Thermo Fisher C7025) or CellTracker Red CMTPX (Thermo FisherC34552) was added in serum free media to the final concentration of 5 µM. Cells were then incubated for 15–45 min in the cell at 37 °C after which they were washed thoroughly 3 × and then proceeded for further processing. Gels are washed once with PBS to remove the media then fixed with 4% Paraformaldehyde for 20 min at room temperature. Gels were then washed two times for 10 min with 100 mM Glycine solution in PBS (PBS–Glycine). Permeabilization was carried out for 30 min with 0.5% Triton after which the gels were washed two times for 10 min with PBS-Glycine. Gels were then blocked with 10% Goat Serum for 3 h. Primary antibody (Phospho Histone (H2A.X) Ser139 (Thermo Fisher MA1-2022), Collagen 1 (Thermo Fisher PA1-26204), Ki67 (Thermo Fisher MA5-14520) 1:250) was incubated in 10% Goat serum in Immunofluorescence Wash solution (PBS + 0.2% Triton + 0.2% Tween 20) overnight at 4 °C. Gels were washed three times prior to secondary antibody Alexa Fluor 555 or 647 secondary antibody (1:1000) along with DNA dye NucBlue (Thermo Fisher R37606) for 3 h or overnight at 4 °C.

### Microscopy and image analysis

The 3D culture samples were scanned with a 10X air objective and 40 × silicone immersion objective. Images were analyzed using custom codes written in Python.

### Statistical testing

All statistical tests are explicitly mentioned, whenever p-values are reported, i.e., in the main text or as part of the figure legends. All reported p-values were adjusted for multiple testing using Bonferroni correction and adjusted p-values < 0.05 were assessed as statistically significant.

### Supplementary Information

Supplementary Figures.

## Data Availability

The authors declare that all data supporting the findings of this study are available within the article and its Supplementary Information or from the corresponding author, G.V.S., upon reasonable request.

## Code availability

Code containing the image processing scripts will be made available in our lab’s GitHub page.
