# Supplementary material for: Detecting radio- and chemoresistant cells in 3D cancer co-cultures using chromatin biomarkers
Source: Sci Rep. 2023 Nov 24;13:20662. doi: 10.1038/s41598-023-47287-2 (PMC10673941; doi:10.1038/s41598-023-47287-2)
Supplement: Supplementary file 1 — Supplementary Figures. [file 41598_2023_47287_MOESM1_ESM.docx]

**Supplementary Information**

**Figure S1. Methods of spheroid growth and cellular/actin quantification per spheroid:** (A) Representative images of spheroids one day after culture on micropatterns. Cells are stained for F-Actin (green) and DNA (blue). (B) Performance of our 3D nuclear segmentation. (C) Quantification of the number of nuclei per sphere in co-cultures. (D) Quantification of the average spheroid size in the co-cultures. (E) Linear Regression between the number of nuclei in a spheroid and the spheroid area. (F) Estimated cell number per co-culture experiment. (G) Representative brightfield image used to estimate cell number in any given 3D co-culture. (H) Workflow for measuring the actin spread beyond the spheroids for representative images of gels containing A431 spheroids and A431/CCKBR spheroids.

**Figure S2. Radioactivity uptake of 3D spheroids:** (A) Cellular uptake shown as percent of total activity of radiolabeled minigastrin analogue [^177^Lu]Lu-PP-F11N after 2 and 4 hours internalization time. Specific activities of the radioligand were 7.3, 24.3 and 73 kBq/pmol. The radioligand was used to treat 3D co-cultures containing HMF3A fibroblasts and A431/CCKBR cells at the absence or presence of blocking peptide, and control untransfected A431 cells, as indicated. The percentage in the red bars indicate CCKBR-specific uptake. (B) HPLC chromatogram indicating 99% radiochemical purity of Lu‐177-labeled PP‐F11N used in this study (cpm; counts per minute). (C) The mean radioactivity of 3D cultures containing untransfected (blue) and CCKBR-overexpressing (red) A431 cells 2 and 20 hours after treatment with 5 MBq/ml of [^177^Lu]Lu-PP-F11N.

**Figure S3. 3D in-vitro skin cancer model with various primary dermal cell lines:** (A) Representative images of the cultures 2 and 24 hours after treatment. Nuclei are labeled with DAPI and fibroblasts are labeled with CellTrackerGreen (scale bar: 200 μm) (B) The fold change in the median spheroid area (20h post-treatment/2h post-treatment) in a gel for A431 cells with or without CCKBR overexpression (abbreviated as A and AC respectively) when co-cultured with two different primary dermal fibroblast lines: fibroblasts isolated from a young (indicated by the postfix “_Y”) individual (GM09503 cell line) and fibroblasts isolated from an older (indicated by the posfix “_O”) individual (GM08401 cell line). When co-cultured with both cell lines (young and old), spheroids display the same behavior: without CCKBR overexpression, spheroids grow between 2h and 20h, while spheroids with CCKBR overexpression shrink between 2h and 20 h. (C) The change in the number of spheroids in a gel for young and old fibroblasts co-cultured with A431 cells (A_Y and A_O respectively) and young and old fibroblasts co-cultured with A431/CCKBR cells (AC_Y and AC_O respectively). When co-cultured with both fibroblast cell lines, spheroids display the same behavior that A431 spheroids have less spheroids at 20h compared to 2h due to spheroid merging, while A431/CCKBR spheroids have more spheroids at 20h compared to 2h due to fracturing.

**Figure S4. Identifying cell types within mixed co-culture spheroids:** (A) There is a significantly higher fraction of ɣH2AX-positive cells in A431/CCKBR spheroids 20h after radiolabeled minigastrin treatment compared to untreated control spheroids (p=0.0003, Welch’s T-Test, n=78 spheroids), indicating that radiotherapy induces DNA damage. (B) There is a significantly higher fraction of DRAQ7-positive cells in A431/CCKBR spheroids 20h after radiolabeled minigastrin treatment compared to untreated control spheroids (p=7.18x10^-7^, Welch’s T-Test, n=78 spheroids), indicating that radiotherapy induces cell death. (C) The radioactivity of 3D co-cultures containing HMF3A fibroblasts and either A431/CCKBR cells (red), control untransfected A431 cells (blue), or mixed A431 and A431/CCKBR cells (red striped bar) 20h after treatment with 5 MBq/ml of [^177^Lu]-PP-F11N. Results show that radioactivity increases with the fraction of the spheroid that is comprised of A431/CCKBR cells. (D) The performance of our cell labelling technique to separate A431 cells from A431/CCKBR cells in mixed spheroids. A431 cells are labeled with CellTracker Green. Images of CellTracker Green is thresholded using Otsu’s threshold and the fraction of the nucleus which contains CellTracker Green is computed. The centroids of the cells are marked in four representative images, labeled by their classified cell type.

**Figure S5. Using nuclear features to predict therapy outcomes:** (A) A scatter plot of ɣH2AX intensity against DRAQ7 intensity for untreated A431/CCKBR co-culture spheroids, used to identify thresholds for the subpopulations. Each datapoint represents a single nucleus (n=3270 nuclei). To demarcate the subpopulations, we identify thresholds based on the 90th percentile of DRAQ7 and ɣH2AX intensity levels in these untreated A431/CCKBR nuclei. The black solid lines denote these thresholds. (B) The abundance of the subpopulations in untreated and treated conditions for 3 biological replicates, n>1600 nuclei per replicate. (C) A confusion matrix demonstrating the performance of a Random Forest classifier at distinguishing the subpopulations based on nuclear shape features and chromatin features alone (excluding ɣH2AX and DRAQ7 intensities) for only cells treated with radiotherapy. The matrix represents the balanced accuracy after 5-fold cross validation, n>100 nuclei per subpopulation. The Random Forest classifier has an accuracy of 54% at identifying the subpopulation that each nucleus belongs to based on the nuclear shape and chromatin features, which is more than 2-fold higher than random chance (25%). (D) 2D projected nuclear area of the subpopulations of the treated group. (E) The ratio of the 80th percentile of DAPI intensity to the 20th percentile of DAPI intensity per nucleus, which is a measure of the heterochromatin-to-euchromatin ratio. Data represents nuclei from treated spheroids only.
